# The impact and safety of encapsulated nanomaterials as a new alternative against carbapenem resistant bacteria. a systematic review

**DOI:** 10.1007/s11274-024-03894-3

**Published:** 2024-01-18

**Authors:** Omnia M. Abdallah, Heba R. Shebl, Eman Abdelsalam, Shereen I. Mehrez

**Affiliations:** 1 Microbiology Department, Faculty of Dentistry, Misr International University, Cairo, Egypt; 2 Pharmacology Department, faculty of Dentistry, Misr International University, Cairo, Egypt

**Keywords:** Animal studies, Antibiotic resistance, Cytotoxicity, Multi drug resistant bacteria, Nanomaterial, Preclinical.

## Abstract

The emergence of multi drug resistant bacterial infections has caused a critical problem with implication on hospitalization and mortality rates. This systematic review aims to review the combined antimicrobial effect of nanoparticles attached to the traditionally used antibiotics, to overcome the antibiotic resistance crisis. In this systematic search we focused on preclinical studies that have used animal models, to test and evaluate the effect of nanomaterials added to antibiotics against gram negative bacteria with carbapenem resistance. Where, this newly formed structure has led to significant decrease in bacterial load in animal model serum. Furthermore, by evaluating nanomaterial cytotoxicity and inflammatory markers, promising results were established, where low toxicity indices were presented, supporting the ability of this new pathway to be used as an alternative to abused antibiotics. Our research collected the various data and showed encouraging preclinical one for using nanomaterials with antibiotics. This undeniable route should be considered, due to its ability to contribute to the treatment of multi drug resistant bacterial infections. These findings provide base for future studies and reinforce the need for more evaluation and testing on the safety of nanomaterials against bacterial infections.

## Introduction

Different bacteria have been subjected to long term usage of antibiotics, leading to the inevitable consequences, where multi drug resistant (MDR) and/or extremely drug resistant (XDR) genes have been acquired by various bacterial species. Multiple Gram positive and Gram negative bacteria have been labelled insensitive to different and recent antibiotics, causing significant increase in mortality rates due to MDR bacterial infections (Yang et al. 2020).

DeLeo et al. (2014) asserted that “10 million people per year will die due to antibiotic multi drug resistant infections and the mortality rate by anti-microbial resistance (AMR) will be more than cancer”. This statement was due to evolution of antibiotic resistant bacteria that has caused limitation to various therapeutic options presented that cannot keep up with the prevalence of carbapenem-resistant Enterobacteriaceae (CRE), carbapenem-resistant *Acinetobacter baumannii* (CRAB), and carbapenem-resistant *Pseudomonas aeruginosa* (CRPA) (Abdelkader et al. 2017; Bateman et al. 2016; Park et al. 2022).

Carbapenems are β-lactam antibiotics that can be considered the last line of defence against MDR bacteria, such as *P. aeruginosa*, *Acinetobacter baumannii*, and *Klebsiella* species (Tiwari et al. 2019). Unfortunately, carbapenemase-producing organisms have acquired resistance against this group of antibiotics (El-Telbany et al. 2022). Antibiotics used against these MDR infections have been inactive, including imipenem and colistin, which were considered among the most potent antibiotics ever existed against severe bacterial infections. Alas, this reputation is fading out, due to the emergence of a very potent carbapenem-resistant *Acinetobacter baumannii* (CRAB), this microbial species has restrained the antibiotic capabilities and caused formation of antibiotic resistant biofilm (Harding et al. 2017; Li et al. 2021; Park et al. 2022).

World health organization (WHO) in 2017 and centre for disease control and prevention (CDC) in 2013, have announced that carbapenem and third-generation cephalosporin resistant *Enterobacteriaceae* are a critical group of bacteria that need scientific focus to create alternative routes in our fight against their resistance to all existing antibiotics. This alert has specified carbapenem-resistant *Acinetobacter baumannii* (CRAB), *P. aeruginosa* and *K. pneumoniae* infections, and their future impact on our life, and listed them as the most serious pathogens that need to be considered and favoured in future research (WHO, 2017) (Goodman et al. 2018; Li et al. 2021). The incidence of multidrug-resistant *A. baumannii* is approximately four times higher than that of multidrug-resistant *Klebsiella pneumoniae* and *Pseudomonas aeruginosa*. This significant increase in infection has caused the CDC to define it as a global threat, induce public health surveillance and preventive actions (Goodman et al. 2018; Li et al. 2021). To further understand the extent of this MDR bacteria, we need to know that *Acinetobacter baumannii* (AB) is a nosocomial pathogen and one of the major causes of death in hospitals. AB has caused various types of infections at different sites such as respiratory tract, skin, eyes, urinary tract, blood, and surgical site. Furthermore, AB is accounted for numerous healthcare-associated infections (HCAIs) in the United States, Europe and China (Li et al. 2021). This type of bacterial infection has caused high mortality rates due to pneumonia and haemodialysis associated infection, that reached up to 60% and 43.4%, respectively (Li et al. 2021). This pathogen high resistance rate against carbapenem antibiotics, has impacted the traditionally used treatment regimen and caused extreme difficulties to eradicate it. The traditional way of treatment for *A. baumannii* infection, is the usage of different antibiotics such as polymyxins, tigecycline, tetracyclines and aminoglycosides (El Zowalaty et al. 2015). These antibiotics can be used alone or combined. The ability of CRAB to resist antibiotic can be referred to its ability to produce carbapenemase enzyme, or alter the antibiotic binding site or cause outer membrane disruption or active efflux of the antibiotic (Verma et al. 2022). Furthermore, this resistance has extended to *K. pneumoniae*, which has higher prevalence than any other *Enterobacteriaceae* member. It is well known for its potency and abilities to cause multiple complications in compromised patients, such as patients with asthma, emphysema, or cystic fibrosis.

Another Gram negative resistant bacteria is *P. aeruginosa*, that can be described as a machiavellian MDR pathogen. This bacterial species has severe threating capabilities to human health worldwide, as it can be found throughout mucosal surfaces and biomedical invasive devices, causing biofilm formation, which is more resistant to antibiotics and more difficult to eliminate (El-Telbany et al. 2022).

All these abilities and severity of MDR infections have created the sense of urgency for finding new alternatives to the inactive antibiotics in our fight against these life threating pathogens (Bateman et al. 2016; Li et al. 2021; Wu et al. 2017; Yang et al. 2020). The ability of MDR Gram negative bacteria to evade antibiotics has caused a catastrophic consequence represented by biofilm formation on wound surfaces, sepsis and septic shock resulting from bacterial infections, with a huge financial burden on hospitals and impacted hospitalization (Grijalva et al. 2020; Pant et al. 2021; Sen and Sarkar 2021). Therefore, the need for alternative antimicrobial approaches is essential and manifested by various recent interventions and technological proposals focusing mainly on treating and preventing these carbapenem-resistant pathogen spread, as controlling these manipulative microorganisms is a highly complicated process once infection has established (Cave et al. 2021; Sen and Sarkar 2021).

Antibiotics and their related drug resistance have caused multiple adverse effects that included and not limited to drug toxicity. Therefore, the search for a new therapeutic route with lower challenges and better outcomes is targeted by multiple scientific researches (Murugan and Rangasamy 2022). Recently, nanoparticle-based targeted delivery strategies was represented as a new pathway in treatment of MDR infections, by using less therapeutic dosages of antibiotics leading to lower side effects or combining nanoparticles with various antibiotics, and observing their effect on MDR bacteria (Abdelkader et al. 2017; Lahiri et al. 2022; Qing et al. 2019; Rajivgandhi et al. 2019). Combining antibiotics with nanomaterial has taken many forms, where one of the most used forms is the formation of trapped antimicrobial agent inside biodegradable/biocompatible nanoparticles as a form of encapsulation, whether the nanoparticles engulf the antibiotic or are chemically attached to it. This form was proposed to prolong the drug release rates, solubility and stability.

This systematic review aims to analyse and put a spotlight on the preclinical studies using nanomaterial combined with antibiotics against MDR bacterial infections, in vivo, using animal models, to estimate the role and effect of this combined structure in the fight against carbapenem resistance.

## Methodology

In this systematic review, authors had reported the search results according to the PRISMA abstract checklist. Our protocol registration number at PROSPERO is CRD42023383018. Any change and its reason will be updated and made public through the PROSPERO database.

We systematically searched using keywords such as enterobacteriaceae, nanomaterials, antibiotic resistance, and carbapenem resistance aspects. Literature search databases of PubMed, BioMed and ScienceDirect were used to find related research studies that evaluated the effect of nanomaterial associated or not associated with antibiotics that acted on animal models, this systematic search was done with restriction to english language up to the date of December 6, 2022.

### Inclusion and exclusion criteria

The included studies met the following criteria: (1) animal experiment; (mice, rats and guinea Pig) (2) successfully established bacterial infection with MDR Gram negative bacteria model; (3) the intervention or treatment used Nanoparticles and Nanomaterial in any shape or form; (4) the control group was traditional antibiotic treatment and/or antimicrobial agents; (5) the outcomes included serological tests, histopathological studies and other tests such as polymerase chain reaction (PCR) and survivability tests. The excluded studies met the following criteria: (1) literature reviews, comments, other reviews, and editorials; (2) in vitro studies; (3) animal models other than mice, rats, and guinea Pig; (4) natural products as treatment; (5) studies on human; (6) studies written in languages other than English.

Data extraction and synthesis were tabulated and included general study characters such as authors, year, and paper’s title. And included animal model, nanomaterial type, resistant bacteria species, the control/traditional antibiotic used, the establishment of infection and intervention method, the outcomes that varied as serological tests, PCR, histopathological investigations, and others.

### Quality and risk of bias assessment

The quality of each included study was assessed independently by two reviewers (OM and SM) using SYRCLE’s risk of bias (RoB) tool (The Systematic Review Centre for Laboratory Animal Experimentation) that has been derived from the cochrane’s risk of bias tool for clinical studies, and adapted for animal studies (Hooijmans et al. 2014). This tool uses ten item-checklist where questions were answered by “Yes” (if the question was adequately answered in the selected paper), “No” (if the question was not answered) or “Unclear” (if there is not enough information to answer by yes or no). Based on the answers to these signalling questions, the risk of bias domains was classified as low, high, or unclear. Finally, an overall risk of bias was evaluated and documented.

### Study selection

**Fig. 1:**
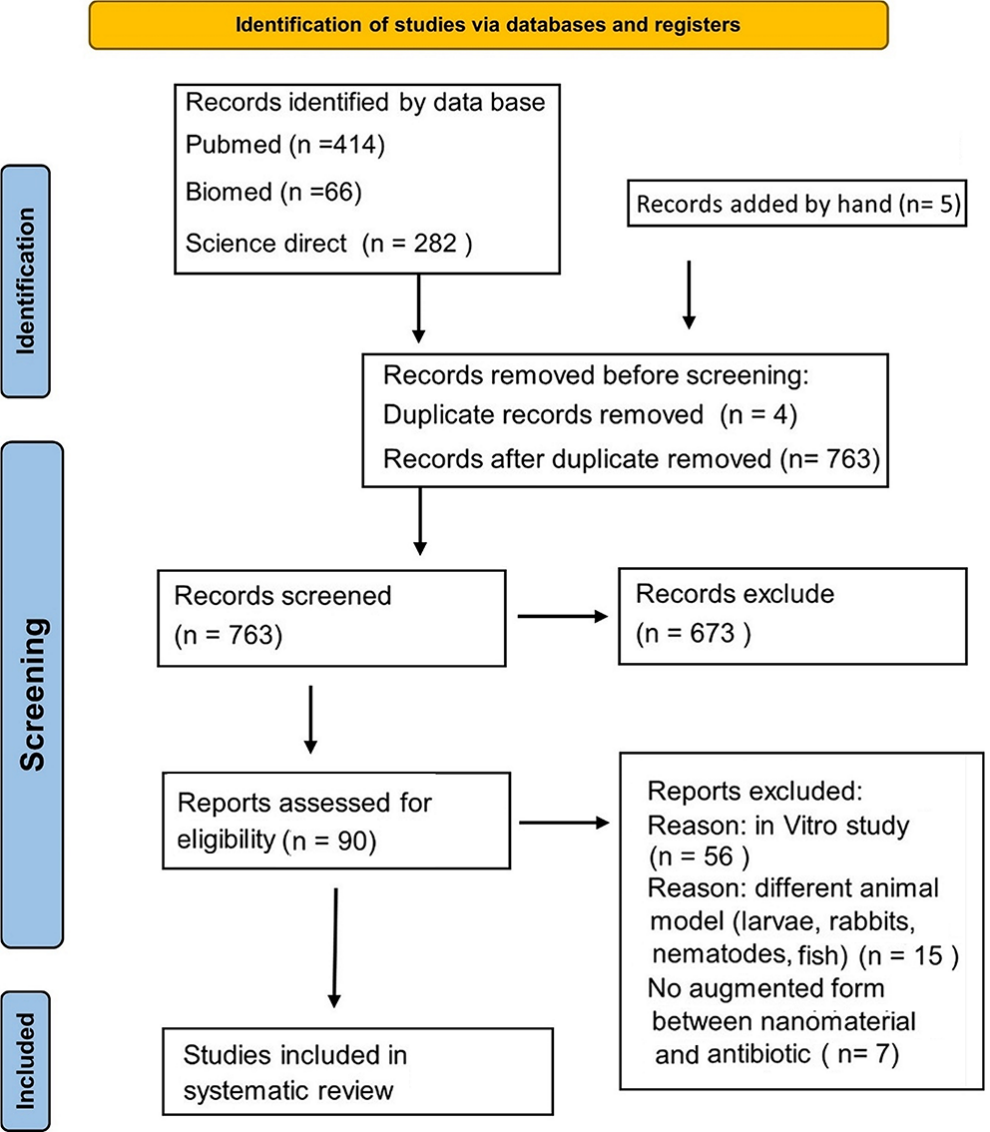
Prisma chart of the study selection process

A total of 763 eligible studies were retrieved from the electronic databases and hand search: 414 from Pubmed, 66 from BioMed and 282 from Science Direct. Finally, Manual search resulted in 5 articles obtained and included. After exclusion due to duplication and abstracts review, 90 articles were selected, where after full reading, 9 studies were included in this systematic review. The process of article selection whether included or excluded is summarized in Fig. 1.

### Characteristics of included studies

All relevant information related to data characteristics and synthesis are tabulated (Table 1). All included studies were in vivo animal studies, published between 2011 and 2022. In these eleven years, only 9 studies were found that were published investigating the combination between nanomaterial and antibiotics using the selected animal model, where 7 studies used mice ( Lam et al. 2016; Li et al. 2021; Liu et al. 2018; Qing et al. 2019; Rishi et al. 2015; Wan et al. 2016a; Yang et al. 2020) and 2 studies used rats (Abdel-Aziz et al. 2017; El-Telbany et al. 2022). Regarding the MDR bacterial isolate used, we found that majority of the selected studies focused on *Acinetobacter baumannii*, referring to their high antimicrobial resistance and their high global health hazard status. Only 2 studies used MDR *Pseudomonas aeruginosa*. In addition, there was one study for each of the other included Gram negative bacteria *Salmonella Typhimurium, Klebsiella pneumoniae*, and *E. coli.*

**Table 1:** The 9 included studies: main characteristics, objectives and results

Author	Title	Animal model	Nanomaterial type	Resistant Bacteria	Control/Traditional antibiotic	Infection establishment	Nanomaterial Administration	Results Outcomes			Cytotoxicity tests
								Serological	Histopathology	Other tests	
(Yang et al. 2020)	Hyaluronic acid and antimicrobial peptide-modified gold/silver hybrid nanocages to combat bacterial multidrug resistance	Specific-pathogen free (SPF) BALB/c mice	HA-P(Au/Ag) HA peptide nanocage	(MDR-AB)	Amikacin	Pneumonia in mice by Intratracheal application of 50 µL, 3 × 10^9^ CFU/mL of MDR-AB	Injected on the second day after infection via the tail vein every day for 3 days	Inflammatory markers, kidney and liver functions, hemolysis test	Survivability rates	X	No specific cytotoxicity tests were performed
(Abdel-Aziz et al. 2017)	Control of imipenem resistant-*Klebsiella pneumoniae* pulmonary infection by oral treatment using a combination of mycosynthesized Ag-nanoparticles and imipenem	Wister albino rats Male (160–180 g)	Spherical AgNPs	Imipenem resistant *K. pneumoniae* (RCMB 5621)	Negative control group (NC) was injected with saline, The second group was the positive control group (PC) infected not untreated, the third group injected orally every day with 0.1 ml of imipenem	Intratracheal application of 0.1 ml (1 × 10^8^ CFU/ml) of imipenem resistant *K. pneumoniae*	variable concentrations of NPs injected orally every day	Blood bacterial counts (CFU/mL) in lungs	Histological analysis with images stained by H&E	TEM	No specific cytotoxicity tests were performed
(Rishi et al. 2015)	Improved oral therapeutic potential of nanoencapsulated cryptdin formulation against *Salmonella* infection	BALB/c mice males (18–22 g) (4–5 weeks old)	Cryptdin-chitosan tripolyphosphate (C-TPP) nanoparticles	*Salmonella enterica serovar Typhimurium* NCTC74	Untreated control group	Mice were infected orally with 0.25 mL of 2.5 × 10^7^ CFU/ mL	nanoparticle formulations administered as a single dose/mouse by oral gavage.	Blood bacterial counts (CFU/mL) in organs (liver and intestine)	Histolopathological evaluation and survivability rates	X	Oxidative damage markers
(Qing et al. 2019)	Thermo-responsive triple-function nanotransporter for efficient chemo-photothermal therapy of multidrug-resistant bacterial infection	BALB/c mice female (6- to 8-week- old)	Imipenem-encapsulated TRIDENT nanoparticles (Thermo-Responsive-Inspired Drug-Delivery Nano-Transporter)	Clinically isolated multidrug-resistant *E. coli* (MDREC)	Imipenem	Subcutaneous injection at the right mice flank, inoculated with 1 × 10^6^ to 1 × 10^7^ CFU mL^− 1^, 100 µl) of MDREC	Imipenem-encapsulated TRIDENT nanoparticles were subcutaneously injected twice	Analysis of sepsis biomarkers	Histopathology analysis of Wound Healing and improvement	X	Biosafety assessment for renal function markers
(Liu et al. 2018)	Supramolecular nanofibers self-assembled from cationic small molecules derived from repurposed poly (ethylene teraphthalate) for antibiotic delivery	Adult C57BL/6 mice (18–22 g) (8-week-old,)	piperacillin/tazobactam (PT) Supramolecular nanofibers	*P. aeruginosa*	Piperacillin/ tazobactam and saline	8 mm in length wounds were made into the dermis and inoculated with 10 µL of *P. aeruginosa* (~ 3 × 10^8^ CFU/ mL)	Administered once daily at an equal dosage for 2 days	Blood bacterial counts (CFU/mL) in tissues	Histopathology of Wound Healing and improvement	X	No specific cytotoxicity tests were performed
(Lam et al. 2016)	Combating multidrug-resistant Gram-negative bacteria with structurally nanoengineered antimicrobial peptide polymers	C57BL/6 mice female (10 to 14-week-old) (23.2 ± 1.7 g)	structurally nanoengineered antimicrobial peptide polymers’ (SNAPPs)	Colistin and multi-drug resistant (CMDR) *A. baumannii* wild-type *A. baumannii* (ATCC 19,606) or CMDR *A. baumannii* (FADDI-AB056)	Imipenem and untreated control groups	Peritonitis infection	Intraperitoneal injected (SNAPPs) (8.3 mg kg^–1^ /dose)	Blood bacterial counts (CFU/mL) in blood (Blood-Cleansing Treatment))	X	X	No specific cytotoxicity tests were performed
(Li et al. 2021)	Novel Multifunctional Silver Nanocomposite Serves as a Resistance-Reversal Agent to Synergistically Combat Carbapenem-Resistant *Acinetobacter baumannii*	BALB/c mice female (Six-week-old)	IPM@AgNPs-PEG-NOTA nanocomposite (silver nanoparticles were coated with SH-PEG-NOTA as well as loaded by imipenem)	Carbapenem-resistant *A. baumannii*	Imipenem	Micro tracheal injection of CRAB by 100 µL (1 × 10^6^ CFU/L	Injected with 200 µL of nanomaterial once a day for 3 consecutive days.	Blood bacterial counts (CFU/mL) in blood (Blood-Cleansing Treatment) inflammatory response and markers	Histopathology analysis of Wound Healing and improvement	X	biosafety assessment of renal function markers
(El-Telbany et al. 2022)	Combination of Meropenem and Zinc Oxide Nanoparticles; Antimicrobial Synergism, Exaggerated Antibiofilm Activity, and Efficient Therapeutic Strategy against Bacterial Keratitis	Adult Sprague-Dawley rats male (220 ± 20 g)	Zinc Oxide Nanoparticles	*P. aeruginosa* PU15	Meropenem	10 µL suspension containing (5 × 10^8^ CFU) of *P. aeruginosa* onto the wounded cornea	ZnO-NPs was administrated two days after infection.	X	X	antibacterial activity against infected cornea	No specific cytotoxicity tests were performed
(Wan et al., 2016)	Effects of silver nanoparticles in combination with antibiotics on the resistant bacteria *Acinetobacter baumannii*	C57BL/6 mice male Six-week-old (body weight ~ 20 g)	AgNPs	*A. baumannii*	Untreated control	Intraperitoneal injection of 5 × 10^9^*A. baumannii*	Intraperitoneal injections of 100 µL total volume.	Inflammatory markers and Blood bacterial counts (CFU/mL) in blood (Blood-Cleansing Treatment)	x	Survival assays and Bacterial colonization assays	No specific cytotoxicity tests were performed

**Abbreviations: AgNPs**: Silver nanoparticles, **MDREC**: Multidrug-resistant *Escherichia coli*, **CRAB**: carbapenem-resistant *A. baumannii*, **CFU**: colony-forming unit

### The combined structures and their augmented properties

Various types of encapsulated nanomaterials were used, and their characteristics were tabulated (Table 1). This diversity highlights the absence of a standardized method with high effectivity. This emerging side of the MDR treatment is new and still under investigation using different types of nanomaterials. Until 2022, only four papers used silver (Abdel-Aziz et al. 2017; Wan et al., 2016; Li et al. 2021) and only one used zinc oxide (El-Telbany et al. 2022). Hundreds of research studies have used silver nanoparticles combined with antibiotics, but these papers targeted the in vitro phase only and did not show the effect of these combined forms on animal models. This gap of knowledge has led to the small number of studies that we have selected from the data base.

Six studies used biodegradable encapsulating nanomaterials such as chitosan, hyaluronic acid, nanofibers, and peptides to be combined with the antibiotic. Several papers showed a common method of augmentation which is the encapsulation, where nanoparticles are used to trap/engulf the antibiotic temporarily, and then release it continuously at the site of infection. This form of encapsulation is a cage-like form, which may be opened or disintegrated at the targeted site; in this way the amount of nanomaterial or antibiotic trapped are not wasted or mistargeted. This leads to usage of lower amounts of antibiotics or nanomaterials with positive effects on cytotoxicity levels and antimicrobial effectivity at lower ranges of therapeutic agents (Lam et al. 2016; Liu et al. 2018; Li et al. 2021; Qing et al. 2019; Rishi et al. 2015; Yang et al. 2020). Another form of encapsulation involves the chemical interaction between nanoparticles or their oxide form with the antibiotics. This route was favoured by other researches, where their antimicrobial efficacy was tested (Abdel-Aziz et al. 2017; El-Telbany et al. 2022; Wan et al., 2016; Wu et al. 2017). We believe that this can be the ideal state for the combined structure, as nanomaterials are in their optimum condition do function as delivery system for the antibiotics and the entire dosage can reach its targeted site. Here we can state that this serves as the optimum form of synergy.

During our research other in vivo studies have used this encapsulation method but without any antibiotic, where they focused on the antimicrobial activity of the nanomaterials alone without any antibiotic additions. This form of studies presents another alternative, where they completely replace the antibiotics by nanomaterials and test their cytotoxicity on animal models. These studies were not selected, as they lack the augmentation step with antibiotics. However, their results cannot be ignored, where they have showed very low cytotoxicity rates and highly promising antimicrobial effectivity (Rasha et al. 2021; Rasha, Monerah, Rasha et al. 2021a, b; Sen and Sarkar 2021).

### Reported outcomes and main results

All relevant selected 9 studies have reported in vivo outcomes using animal models. The measured outcomes included serological evaluations and/or histopathological examination from infected and treated animal models (Table 2). In addition, other variable outcomes such as bioluminescence imaging (luminescence images of the infected wound area) and/or survival assays were reported. Here we found that eight of the selected (9) studies reported serological results measuring the effect of the nanomaterial used against the blood bacterial counts (colony forming unit: CFU/mL) in blood (blood-cleansing treatment) of the animal model used and/or the inflammatory responses and markers of kidney and liver functions of infected and treated animal models (Lam et al. 2016; Li et al. 2021; Liu et al. 2018; Qing et al. 2019; Wan et al., 2016; Yang et al. 2020 Rishi et al. 2015). Only El-Telbany et al. (2022) used a combined structure of meropenem–ZnO-NPs combination to treat localized inflected *P. aeruginosa* infection in the corneas of animal models. They showed the ability of nanoparticles to carry the antibiotic beyond its realm and target specifically the area of infection. The results showed significantly; than those obtained from using antibiotics alone. However, this study did not evaluate the capabilities of the nanomaterial alone or its efficacy without antibiotics, which may be seen as biassed attribution to the combined forms.

**Table 2:** Augmented structures effect on animal models infected by MDR bacteria

Author	Survivability rates	Bacterial Clearance	Inflammatory markers	Kidney & Liver Functions	Histopathological Results
(Yang et al. 2020)	100%	HAP(Au/Ag)/NIR treatment inhibited MDR-AB and showed the least CFU	Significant decrease in levels of TNF-α and IL-6 (220.74 pg/mL and 153.07 pg/mL, respectively)	UREA, CREA, ALT, and AST values were similar to that of normal mice (7mmoL/L, 13µmoL/L, 32U/L, and 173U/L, respectively)	Treatment showed 1) recovered separated pulmonary alveoli structures, like normal lung tissue. 2) Red blood cells around alveolar walls were significantly reduced.
(Abdel-Aziz et al. 2017)	cured lung cell after treatment (after 14 days) with no survivability percentage	complete eradication of bacterial infection	No inflammatory markers tested	No kidney and liver functions detected	Treatment showed 1) Presence of classically activated macrophages in lung tissues that help in host defence
(Rishi et al. 2015)	survivability 87% (after 21 days)	Liver: Reduction in CFU by 1.6- 2 log Intestine: Reduction in CFU by 1.7- 2 log	x	liver tissues: 1) nitrite levels decreased to 4 micromoles/mg protein. 2) catalase increased by 2.4-fold	Treatment showed: 1) Small intestine sections revealed decreased infiltration of lymphocytes, normal ileum. 2) liver tissue showed decrease in infiltration of lymphocytes. with recovery from damage to normal liver cells.
(Qing et al. 2019)	No survivability rates (on skin infection)	70% decrease in skin lesion size	PCT and CRP were measured against Gram positive MRSA-infected mice only (the results did not include Gram negative, MDREC)	BUN less than 15 mmol/L and creatinine less than 8 µmol/L	Skin tissues intact histological dermis structures
(Liu et al. 2018)	No survivability rates (on skin infection)	Reduction in CFU by 1.3 log	x	x	Skin tissue: increase in epidermal thickness, and significant reduction in inflammatory infiltrate
(Lam et al. 2016)	100%	Peritoneal cavity: Reduction in CFU by > 5-log Spleen: Reduction in CFU by > 3-log	x	x	x
(Li et al. 2021)	100% (After 1 week)	Reduction in CFU by 4-log	significant decrease in CRP (4.76 ± 1.94) and IL-6 and TNF-α ≈ 30 pg/ mL	ALT, AST, BUN and CREA, values were similar to that of normal mice. (113.6 ± 16.1 U/L, 121.4 ± 24.5U/L, 7.52 ± 2.04 mmol/L, 14.67 ± 2.81 mmol/L, respectively)	Reduced inflammatory infiltration debris with no alveolar walls thickening or congestion
(El-Telbany et al. 2022)	No survivability (induced inflammatory keratitis) (cornea of the eye)	Smallest area % of corneal opacity (less than 10%)	x	x	x
(Wan et al. 2016a)	Survivability 60%	complete eradication of bacterial infection	Not tested as augmented structure (measured against antibiotic and nanomaterial, separately)	Recorded only in *vitro*	x

**Abbreviations: AgNPs**: Silver nanoparticles, **HA-P(Au/Ag)/NIR**: Hyaluronic acid and peptide-modified (gold/silver)/ near-infrared, **MDR-AB**: Multi-drug Resistant *Acinetobacter baumannii*, **SNAPPs**: Structurally nanoengineered antimicrobial peptide polymers, **IPM@AgNPs-PEG-NOTA**: imipenem, silver nanoparticles- Poly(ethylene glycol)- triazacyclononane-1,4,7-triacetic acid, **TRIDENT**: Thermo-Responsive-Inspired Drug-Delivery Nano-Transporter, **PT**: piperacillin/tazobactam, **MDREC**: Multidrug-*resistant Escherichia coli*, **CRAB**: carbapenem-resistant *A. baumannii*, **CFU**: colony-forming unit, **PMB**: Polymyxin B, **PCT**: procalcitonin, **CRP**: c-reactive protein, **CREA**: creatinine, **ALT**: alanine transaminase, **AST**: aspartate amino-transferase, **BUN**: blood urea nitrogen, **CREA**: creatinine, **pg/mL**: picograms per millilitre, **mmoL/L**: Millimoles per liter, **µmoL/L**: Micromole per liter, **U/L**: Units per litre, **µg**: Microgram, **µM**: micrometre

Six studies used histological assessment to verify the effectivity of nanomaterial associated with antibiotics, on the architecture of different organ tissue and calculate the count of inflammatory cells, beside the evaluation of the inflammatory response (Abdel-Aziz et al. 2017; Li et al. 2021; Liu et al. 2018; Qing et al. 2019; Rishi et al. 2015; Yang et al. 2020).

Only 3 studies extensively tested and discussed the cytotoxic effect of nanomaterials using cryptdin-chitosan tripolyphosphate (C-TPP) nanoparticles, imipenem-encapsulated TRIDENT nanoparticles (Thermo-Responsive-Inspired Drug-Delivery Nano-Transporter) and IPM@AgNPs-PEG-NOTA nanocomposite (silver nanoparticles were coated with SH-PEG-NOTA as well as loaded by imipenem). These three relatively new nanomaterials have been tested for their biosafety on renal functions and histological analysis for five major organs (heart, liver, spleen, lung, and kidney) (Li et al. 2021; Qing et al. 2019; Rishi et al. 2015). One of the most important results was recorded by one of these studies, where Rishi et al. (2015) evaluated the effect of nanocapsulated cryptdin on small intestine and liver tissues of mice as animal model. This new nano form of encapsulation has showed its biocompatibility with healing effects on liver cells and intestine tissues that recovered normally after the infection. This study showed that the nanoencapsulated combined structure showed a significant decrease in MDA (malomdialdehdye) and nitrate levels, and increased the concentration of catalase enzyme and superoxide dismutase (SOD) activity, which positively impacted the capability of the animal model immune system, as the host tissues were able to scavenge superoxide radicals generated by the burst of immune cells, in comparison to cryptdin-only treated group. These findings further strengthen the impact of nanosized material on the penetration power of the drug and their effectivity and reachability. This effect can only be achieved by the nanosized materials that are able to penetrate targeted sites.

Other studies showed the proinflammatory cytokines in the animal plasma, lung tissues and the levels of tumour necrosis factor alpha (TNF-α) and interleukin (IL)-6, where this combined encapsulated form had the lowest concentration of biomarkers and enhanced the antimicrobial activity of the antibiotic, whether the infection was established by MDR *Acinetobacter baumannii*, or *K. pneumoniae* (Abdel-Aziz et al. 2017; Li et al. 2021; Wan et al. 2016a; Yang et al. 2020). Furthermore, by studying the effect of these newly formed encapsulated structures on kidney and liver functions by measuring ALT, AST, UREA and CREA concentrations in animal serum, results indicated minimal blood bacterial counts (CFU/mL) in blood (blood-cleansing treatment) and cytotoxicity with significant increase in mice survivability rates, therefore improving their health overall.

Qing et al. (2019), used combined structures, TRIDENTs delivery system, which were evaluated for its safety and biocompatibility using biomarkers analysis and histological images. These results showed no abnormalities in the hematoxylin and eosin stain (H&E) stained sections of the heart, liver, spleen, lung and kidney. This encapsulation of antibiotics can inhibit imipenem (IMP) degradation and nephrotoxicity caused using cilastatin that is normally co-administered in clinical practices. This new delivery device used the combined structure on another level and showed the future prospect of nano delivery route of antibiotics at small doses.

Liu et al. (2018) used nanomaterial as carrier by coating nanofibers with antibiotics (piperacillin/tazobactam (PT). Results from histological analysis, showed that this coated combined structure exhibited a significant increase in mean epidermal thickness, and induced significant reduction in inflammatory infiltrate, re-epithelization with wide granulation tissue. In addition, sustained antibiotics release has extended the drug half-life, improved its therapeutic index, and optimized its pharmacokinetics profiles.

Two studies have showed the highest survival rates up to 100% (Rishi et al. 2015; Yang et al. 2020). Another important aspect was targeted and discussed in 6 studies, was patients’ compliance, where they showed that using nanoparticles combined with antibiotic, led to lower dosage and frequencies. (Abdel-Aziz et al. 2017; El-Telbany et al. 2022; Li et al. 2021; Liu et al. 2018; Qing et al. 2019; Wan et al., 2016). This aspect should be addresed and highlighted in any upcoming study regrading nanomaterial and antibiotics, as it will affect darstically affect future clinical studies.

### Risk of bias assessment

**Fig. 2:**
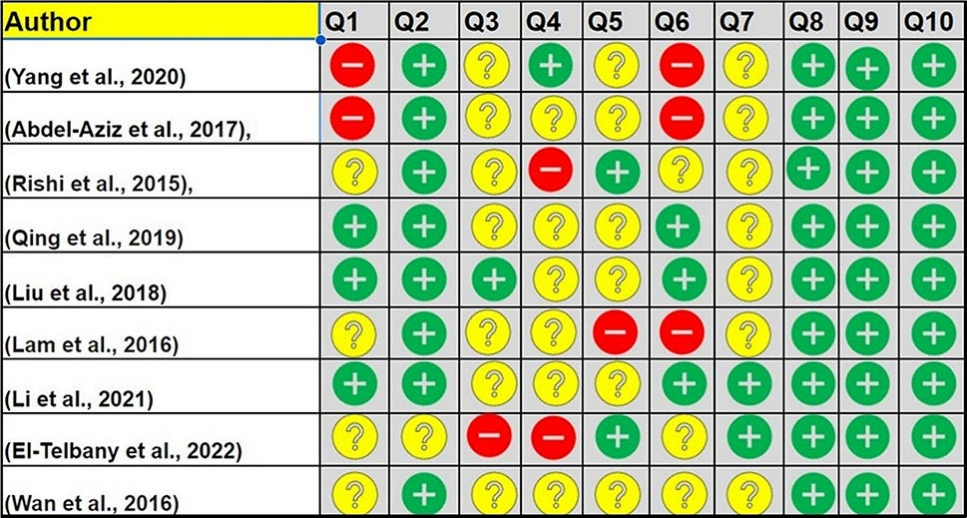
Risk of bias assessment evaluated according to the SYstematic Review Centre for Laboratory Animal Experimentation (SYRCLE): authors’ judgment about each risk of bias item (green = low, yellow = moderate, red = high)

**Fig. 3:**
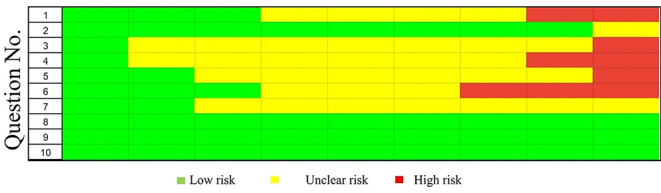
Risk of bias assessment evaluated according to the SYstematic Review Centre for Laboratory Animal Experimentation (SYRCLE): authors’ judgment about each risk of bias item presented as proportions. Selection bias: (1) sequence generation; (2) baseline characteristics; (3) allocation concealment. Performance bias: (4) random housing; (5) blinding. Detection bias: (6) random outcome assessment; (7) blinding. Attrition bias: (8) incomplete outcome data. Reporting bias: (9) selective outcome reporting. 10) Other

Risk of bias assessment was performed using the SYstematic Review Centre for Laboratory Animal Experimentation (SYRCLE), this methodological approach was used to synthesize evidence (Hooijmans et al. 2014). Using this tool has led to a total of 90 entries. Five studies showed some elements of high-risk bias, because of sequence generation and blinding domains. The other 4 studies were assessed as moderate risk of bias, as no item was registered as high risk of bias and most of the items were judged as low to moderate risk of bias. In more detailed assessment, among the 90 entries, 52.22% were answered as “Yes”, 10.0% as “No” and the remaining 37.78% as “Unclear” (Fig. 2). Selection bias sequence generation, baseline characteristics and allocation concealment frequently judged as of low risk of bias, performance bias random housing and blinding were unclear risk of bias, and detection bias random outcome assessment ranged from high risk of bias in some studies and unclear risk of bias other selected studies. Finally, attrition bias, reporting and other bias domains showed majority of low risk of bias results (Fig. 3).

## Discussion

All collected data from studies were compared and analysed as possible. The overall results of the present systematic review showed that augmentation of antibiotics with nanomaterial can help MDR bacteria and help in preventing and controlling emergence of more resistant bacterial infections, which will eventually impact the hospitalization periods and patient recovery process.

Different methods were used in studies to optimize the augmentation state between the nanomaterial and the antibiotic. Where encapsulation was presented in most selected papers, supporting the hypothesis that this form can reduced lung tissue edema and cause no thickening or congestion of the alveolar walls.

These unequivocal results lead to consequential assumption that these new structures are safe, with good biocompatibility that was reflected by the RBCs count of combined treated group and with no impact on body weight of animal model. This can be considered as a new route against MDR bacteria emergence. These studies also have indicated that the antibiotics alone are unable to reach the lung tissues at satisfactory concentrations to stop the MDR bacterial invasion, and therefore alleviate the burden on the internal organs and influence survival rates as indicated in these studies.

One study conducted by Abdelkader et al.(2017), showed that encapsulation of meropenem-loaded chitosan nanoparticles has caused improvement on the survival and bacterial clearance rates compared to antibiotic only. The only problem here, is that they tested this combined structure against meropenem sensitive bacteria, excluding the MDR aspect, causing a speculation about the ability of this form to treat MDR infection, although, this encapsulated meropenem has extended release and sustainability causing more efficient antimicrobial activity and drug penetration against different bacterial cell parts, but all these results were against sensitive bacteria. This can be judged as expected results, as various species of bacteria are susceptible already with antibiotics alone without the need for any other antimicrobial accelerating force.

On the other hands some studies have favoured the option of using the nanomaterial alone (such as graphine oxide, zinc oxide, copper, and silver nanoparticles), without any additions, against different MDR bacterial infections. These studies were excluded from our review as they did not assess the combined augmented structure of nanomaterial and antibiotics. But their results shade some light on the possibility of replacing antibiotics from our equation in the fight against MDR bacteria. These studies have provided evidence, using animal models, that nanomaterials alone can cause antimicrobial destruction to the MDR bacteria and elevate the survival rates more than 80%. They also highlighted, using histopathological images, the ability of these nanomaterials to improve and accelerate wound healing by impacting the skin regeneration at epidermal layer with keratinized covering layer and lowering bacterial inflammation without causing tissue damage, in comparison to antibiotics alone. (Huang et al. 2011; Rasha et al. 2021a, b; Sen and Sarkar 2021; Wu et al. 2017).

Two papers have used synthesized nanodevices, one by Qing et al. (2019), who synthesized spherical shaped nanomaterial TRIDENT (Thermo-Responsive-Inspired Drug-Delivery Nano-Transporter) that showed its ability to facilitate imipenem (IMP) permeability causing irreversible damage to bacterial membranes, causing cell death and preventing sepsis development. The other nanodevice was created by Park et al. (2022), who used magnetic nanovesicles (MNVs) with ability to remove wide range of antibiotic resistant bacteria from rats blood stream, without triggering undesirable immune reactions, as these MVNs used human cell membranes as camouflage. In their paper, they ignored the antibiotic usage completely, by using a cleansing nanodevice attached to anesthetized rats’ jugular veins. The study investigated bacterial load in vital organs (lungs, spleens, and kidneys) and showed significant reduction in CFU values compared to other groups. Furthermore, WBC and platelets count showed normal levels with MVNs treated group. Colistin, the traditional antibiotic used against CR *E.coli*, failed to achieve similar results. This MNVs showed a significant potentiality to function as a nanocarrier with antimicrobial activity against multi drug resistant infections.

All these studies, included and excluded, showed, and explored the efficiency of encapsulation of traditionally used antibiotics and nanomaterials, forming extended structure, or the nanomaterial alone in the fight against MDR infections. However, perhaps the need for an alternative route, has somehow influenced researchers to highlight the pros of nanomaterial and antibiotics together in this fight, and ignored or downplayed the cons of these combined encapsulated structures. This can be seen in the lack of any papers, done on animal selected models, which has shown any kind of toxicity. This alarming observation has pointed us toward the need of more research and studies, on animal subjects, that critically and specifically target the cytotoxicity of encapsulated structure.

## Conclusion

The overall results from all included studies have shown that the augmentation of nanomaterials with antibiotics has a positive effect on the antimicrobial activity of the antibiotic and its biocompatibility. Furthermore, these encapsulated structures, have low cytotoxic results and have positively impacted the survival rates of animal models in preclinical studies. Nanomaterials and antibiotic have high sustainability, with an extended-release rates, which is crucial in the pursuit of alternative, to traditional antibiotics, against MDR bacteria. This systematic review shades some light on the effectivity of this new pathway, in terms of antibiotic activity and controlling the spread of bacterial infection. Additionally, the ability of newly formed structures to deliver the antibiotic carried by nanomaterials to its requested site, is prominent. This preclinical systematic review has used the available studies conducted using encapsulated newly formed structure against MDR bacteria, to provide straightforward evidence of our current status and clarify the need for more preclinical data to the nanosystem route in the treatment of carbapenem MDR bacteria.

Finally, To the best of our knowledge, this is the first systematic review that collects data about the usage of nanomaterials in our future fight against Gram negative MDR bacteria and provide a step that will fill the knowledge gap in our antibiotic resistance challenge and reaffirm the importance of finding a solution for the antibiotic resistance pandemic, by providing a route that should be explored and taken to the next level of research and studies.

## Data Availability

The datasets generated during and/or analysed during the current study are available from the corresponding author on reasonable request.

## References

[CR1] Abdel-Aziz MM, Yosri M, Amin BH (2017). Control of imipenem resistant-*Klebsiella pneumoniae* pulmonary infection by oral treatment using a combination of mycosynthesized Ag-nanoparticles and imipenem. J Radiation Res Appl Sci.

[CR2] Abdelkader A, El-Mokhtar MA, Abdelkader O, Hamad MA, Elsabahy M, El-Gazayerly ON (2017). Ultrahigh antibacterial efficacy of meropenem-loaded chitosan nanoparticles in a septic animal model. Carbohydr Polym.

[CR3] Bateman RM, Sharpe MD, Jagger JE, Ellis CG, Solé-Violán J, López-Rodríguez M, Herrera-Ramos E, Ruíz-Hernández J, Borderías L, Horcajada J, González-Quevedo N, Rajas O, Briones M, de Castro Rodríguez, Rodríguez Gallego F, Esen C, Orhun F, Ozcan GE, Senturk P, Prandi E (2016) E. 36th International Symposium on Intensive Care and Emergency Medicine: Brussels, Belgium. 15–18 March 2016. In *Critical care (London, England) (*Vol. 20, Issue Suppl 2, p. 94). 10.1186/s13054-016-1208-6 10.1186/s13054-016-1208-6 PMC5493079 27885969

[CR4] Cave R, Cole J, Mkrtchyan HV (2021). Surveillance and prevalence of antimicrobial resistant bacteria from public settings within urban built environments: challenges and opportunities for hygiene and infection control. Environ Int.

[CR5] DeLeo FR, Chen L, Porcella SF, Martens CA, Kobayashi SD, Porter AR, Chavda KD, Jacobs MR, Mathema B, Olsen RJ, Bonomo RA, Musser JM, Kreiswirth BN (2014). Molecular dissection of the evolution of carbapenem-resistant multilocus sequence type 258 Klebsiella pneumoniae. Proc Natl Acad Sci USA.

[CR7] El Zowalaty ME, Al Thani AA, Webster TJ, Zowalaty E, Schweizer AE, Nasrallah HP, Marei GK, Ashour HM (2015). *Pseudomonas aeruginosa*: arsenal of resistance mechanisms, decades of changing resistance profiles, and future antimicrobial therapies. Future Microbiol.

[CR6] El-Telbany M, Mohamed AA, Yahya G, Abdelghafar A, Abdel-Halim MS, Saber S, Alfaleh MA, Mohamed AH, Abdelrahman F, Fathey HA, Ali GH, Abdel-Haleem M (2022) Combination of Meropenem and Zinc Oxide nanoparticles; Antimicrobial Synergism, Exaggerated Antibiofilm Activity, and efficient therapeutic strategy against bacterial keratitis. Antibiot (Basel Switzerland) 11(10). 10.3390/antibiotics11101374 10.3390/antibiotics11101374 PMC9598448 36290032

[CR8] Goodman KE, Simner PJ, Klein EY, Kazmi AQ, Gadala A, Rock C, Tamma PD, Cosgrove SE, Maragakis LL, Milstone AM (2018). How frequently are hospitalized patients colonized with carbapenem-resistant *Enterobacteriaceae* (CRE) already on contact precautions for other indications?. Infect Control Hosp Epidemiol.

[CR9] Grijalva M, De La Torre K, Sánchez N (2020). The clinical impact of a multiplex real-time PCR system for microbiological diagnosis of sepsis: a mortality study. New Microbiol.

[CR10] Harding CM, Pulido MR, Di Venanzio G, Kinsella RL, Webb AI, Scott NE, Pachón J, Feldman MF (2017). Pathogenic *Acinetobacter* species have a functional type I secretion system and contact-dependent inhibition systems. J Biol Chem.

[CR11] Hooijmans CR, Rovers MM, De Vries RBM, Leenaars M, Ritskes-Hoitinga M, Langendam MW (2014) SYRCLE’s risk of bias tool for animal studies. BMC Med Res Methodol 14(1). 10.1186/1471-2288-14-43 10.1186/1471-2288-14-43 PMC4230647 24667063

[CR12] Huang L, Dai T, Xuan Y, Tegos GP, Hamblin MR (2011). Synergistic combination of chitosan acetate with nanoparticle silver as a topical antimicrobial: efficacy against bacterial burn infections. Antimicrob Agents Chemother.

[CR13] Lahiri D, Nag M, Dey A, Sarkar T, Pati S, Ray RR (2022) Nanoparticles based antibacterial vaccines: Novel strategy to combat antimicrobial resistance. Process Biochemistry, *119, 82–*89. 10.1016/j.procbio.2022.05.011

[CR14] Lam SJ, O’Brien-Simpson NM, Pantarat N, Sulistio A, Wong EHH, Chen YY, Lenzo JC, Holden JA, Blencowe A, Reynolds EC, Qiao GG (2016) Combating multidrug-resistant Gram-negative bacteria with structurally nanoengineered antimicrobial peptide polymers. Nat Microbiol 1. 10.1038/nmicrobiol.2016.162 10.1038/nmicrobiol.2016.162 27617798

[CR15] Li X, Gui R, Li J, Huang R, Shang Y, Zhao Q, Liu H, Jiang H, Shang X, Wu X, Nie X (2021). Novel Multifunctional Silver Nanocomposite serves as a resistance-reversal Agent to synergistically Combat Carbapenem-Resistant *Acinetobacter baumannii*. ACS Appl Mater Interfaces.

[CR16] Liu S, Fukushima K, Venkataraman S, Hedrick JL, Yang YY (2018). Supramolecular nanofibers self-assembled from cationic small molecules derived from repurposed poly(ethylene teraphthalate) for antibiotic delivery. Nanomedicine: Nanatechnol Biology Med.

[CR17] Murugan D, Rangasamy L (2022). The use of antimicrobial biomaterials as a savior from post-operative vascular graft-related infections: a review. Results in Engineering.

[CR18] Pant A, Mackraj I, Govender T (2021). Advances in sepsis diagnosis and management: a paradigm shift towards nanotechnology. J Biomedical Sci (Vol.

[CR19] Park SJ, Kwon S, Lee MS, Jang BH, Guzmán-Cedillo AE, Kang JH (2022). Human cell-camouflaged Nanomagnetic Scavengers Restore Immune Homeostasis in a Rodent Model with Bacteremia. Small.

[CR20] Qing G, Zhao X, Gong N, Chen J, Li X, Gan Y, Wang Y, Zhang Z, Zhang Y, Guo W, Luo Y, Liang XJ (2019) Thermo-responsive triple-function nanotransporter for efficient chemo-photothermal therapy of multidrug-resistant bacterial infection. Nat Commun 10(1). 10.1038/s41467-019-12313-3 10.1038/s41467-019-12313-3 PMC6760232 31551496

[CR21] Rajivgandhi G, Maruthupandy M, Veeramani T, Quero F, Li W-J (2019). Anti-ESBL investigation of chitosan/silver nanocomposites against carbapenem resistant *Pseudomonas aeruginosa*. Int J Biol Macromol.

[CR22] Rasha E, Alkhulaifi MM, AlOthman M, Khalid I, Doaa E, Alaa K, Awad MA, Abdalla M (2021a) Effects of Zinc Oxide Nanoparticles Synthesized Using Aspergillus niger on Carbapenem-Resistant *Klebsiella pneumonia In Vitro* and *In Vivo*. Frontiers in Cellular and Infection Microbiology, 11, 748739. 10.3389/fcimb.2021.748739 10.3389/fcimb.2021.748739 PMC8635236 34869059

[CR23] Rasha E, Monerah A, Manal A, Rehab A, Mohammed D, Doaa E (2021b) Biosynthesis of Zinc Oxide Nanoparticles from Acacia nilotica (L.) Extract to Overcome Carbapenem-Resistant *Klebsiella Pneumoniae*. Molecules (Basel, Switzerland), 26(7). 10.3390/molecules26071919 10.3390/molecules26071919 PMC8037469 33805514

[CR24] Rishi P, Bhogal A, Arora S, Pandey SK, Verma I, Kaur IP (2015). Improved oral therapeutic potential of nanoencapsulated cryptdin formulation against Salmonella infection. Eur J Pharm Sci.

[CR25] Sen S, Sarkar K (2021) Effective Biocidal and Wound Healing Cogency of Biocompatible glutathione: citrate-capped copper oxide nanoparticles against Multidrug-Resistant Pathogenic Enterobacteria. 27(5):616–627 Microbial Drug Resistance (Larchmont, N.Y.). 10.1089/mdr.2020.0131 10.1089/mdr.2020.0131 33048008

[CR26] Tiwari V, Rajeswari MR, Tiwari M (2019). Proteomic analysis of iron-regulated membrane proteins identify FhuE receptor as a target to inhibit siderophore-mediated iron acquisition in *Acinetobacter baumannii*. Int J Biol Macromol.

[CR27] Verma P, Tiwari M, Tiwari V (2022). Potentiate the activity of current antibiotics by naringin dihydrochalcone targeting the AdeABC efflux pump of multidrug-resistant *Acinetobacter baumannii*. Int J Biol Macromol.

[CR28] Wan G, Ruan L, Yin Y, Yang T, Ge M, Cheng X (2016). Effects of silver nanoparticles in combination with antibiotics on the resistant bacteria *Acinetobacter Baumannii*. Int J Nanomed.

[CR29] Wu X, Tan S, Xing Y, Pu Q, Wu M, Zhao JX (2017). Graphene oxide as an efficient antimicrobial nanomaterial for eradicating multi-drug resistant bacteria *in vitro* and *in vivo*. Colloids Surf B.

[CR30] Yang J, Zhao Y, Cao J, Gong C, Zuo J, Zhang N, Zhao Y (2020). Hyaluronic acid and antimicrobial peptide-modified gold/silver hybrid nanocages to combat bacterial multidrug resistance. Int J Pharm.

